# Influence of Different Lithium Compounds on Hydration and Mechanical Properties of Calcium Sulfoaluminate Cement

**DOI:** 10.3390/ma13163465

**Published:** 2020-08-06

**Authors:** Hongyang Deng, Xuanchun Wei, Shaoyan Liu, Shan Li, Xinhua Cai

**Affiliations:** 1 State Key Laboratory of Water Resources and Hydropower Engineering Science, Wuhan University, Wuhan 430072, China; denghy@whu.edu.cn (H.D.); xuanchun.wei@foxmail.com (X.W.); liushaoyan@whu.edu.cn (S.L.); 2 School of Civil Engineering, Wuhan University, Wuhan 430072, China; lishan@whu.edu.cn

**Keywords:** calcium sulfoaluminate (CSA) cement, lithium compounds, hydration, acceleration, mechanical property

## Abstract

This work investigated the influence of three different lithium compounds, lithium carbonate (Li_2_CO_3_), lithium sulfate (Li_2_SO_4_) and lithium chloride (LiCl), on the hydration and mechanical properties of calcium sulfoaluminate (CSA) cement mixtures. Five concentrations of Li^+^, 0, 0.05, 0.11, 0.16 and 0.22 mmol/g of cement, were chosen, and then the proportions (by mass) of three lithium compounds were determined. Compressive strengths at 8 h, 24 h and 28 days were tested. Meanwhile, an early hydration heat test, thermogravimetric (TG) analysis, X-ray diffraction (XRD) and scanning electron microscope (SEM) techniques were performed to study the influences of different lithium compounds on properties of CSA cement mixtures. The experimental results show that three lithium compounds can all accelerate the early hydration process of CSA cement. There is not a remarkable difference on the properties of CSA cement pastes with a different content of Li^+^. The anion of lithium compounds can also affect the properties of CSA cement pastes, the accelerating effects of LiCl and Li_2_SO_4_ are more significant than that of Li_2_CO_3_, but there is not a distinct difference between LiCl and Li_2_SO_4_.

## 1. Introduction

Cement-based materials are the most used artificial materials in the world attributing to their favorable features such as low prices, reliable mechanical properties, high versatilities [1,2,3], etc. As a kind of promising cement-based material [4], calcium sulfoaluminate (CSA) cement was developed in the 1970s [5], and manufactured by calcining natural materials such as limestone, bauxite and clay together with gypsum or anhydrite at 1250–1350 °C, which is lower than Portland cement’s [6]. The main constituents of CSA cement are ye’elimite (3CaO·3Al_2_O_3_·CaSO_4_, $C_{4}A_{3}\bar{S}$), belite (2CaO·SiO_2_, C_2_S) and calcium sulfate (anhydrite, $C\bar{S}$; or gypsum, $C\bar{S}H_{2}$) [7,8,9]. The properties of CSA cement pastes are dominated by ettringite (AFt, $C_{6}A{\bar{S}}_{3}H_{32}$) generated by the hydration of ye’elimite with calcium sulfate, calcium monosulfoaluminate hydrate (AFm, $C_{4}A\bar{S}H_{12}$) formed by the hydration of ye’elimite with water, aluminum hydroxide (AH_3_) and calcium silicate hydrate (C-S-H) [4,10]. In contrast to Portland cement, CSA cement has many advantages, such as low energy consumption during manufacturing, low CO_2_ emission, high early strength development and very good durability under normal service conditions, particularly in marine construction [7,11,12,13]. However, the durability problems caused by strength shrinkage and surface carbonization in the later period are more likely to occur [14,15]. The hydration process of CSA cement can be represented by Equations (1)–(3) [16,17,18].

(1) $$C_{4}A_{3}\bar{S}+2C\bar{S}H_{2}+34H\to AFt+2AH_{3}$$

(2) $$C_{4}A_{3}\bar{S}+18H\to AFm+2AH_{3}$$

(3) $$C_{2}S+nH\to C-S-H+CH$$

In recent years, special materials for emergency repairs are widely used in engineering applications, which require a high early strength [19]. In order to improve the early strength in the very short term, many researchers try to use an early strength agent during the application of cement-based materials. Some studies reported that the hydration degree of cement mortar can be improved by destructing the protective film formed by cement hydration to shorten the induction period, and the small radius of lithium ion has a good destructive effect on the protective film [20,21,22,23,24]. According to the previous studies, it is found that the hydration process of CSA cement can be notably accelerated by lithium carbonate (Li_2_CO_3_) [25], the setting time can be significantly shortened and the early strength can be greatly improved [26]. The early hydration of CSA cement can be accelerated by increasing the dosages of lithium compounds up to 0.03 mmol/g of cement (by the concentration of Li^+^) either in paste or in diluted and stirred suspension, and then levels off [19,27]. When adding lithium hydroxide and sodium borate into CSA cement, lithium ions promoted the initial precipitation of a borated AFm phase, which would later convert into a borated AFt phase [28], and it seems to be no interaction occurred between lithium and borate ions [16,28]. In addition to Li_2_CO_3_, lithium sulfate (Li_2_SO_4_) can also accelerate the hydration reaction of CSA cement, shorten setting time and enhance the strength whether at an early age or a long-term age [29]. However, there are few reports on the effect of lithium chloride (LiCl) on CSA cement.

Although the influence of Li_2_CO_3_ on CSA cement has been widely studied in the literature mentioned above, the acceleration mechanism of Li^+^ and other anions of lithium salts on the hydration of CSA cement is still confusing. This work mainly studied the influence of different anions of three soluble lithium compounds (Li_2_CO_3_, Li_2_SO_4_ and LiCl) on the hydration properties of CSA cement and tried to reveal the mechanism of accelerating effect of lithium compounds, provided support for application of different lithium compounds in CSA cement.

## 2. Experimental

### 2.1. Materials and Preparation of Specimens

A commercial CSA cement, whose chemical composition was shown in Table 1, was mixed with three different kinds of lithium compounds, Li_2_CO_3_, LiCl and Li_2_SO_4_.

The three lithium compounds using in this study were lithium carbonate (Li_2_CO_3_), lithium chloride monohydrate (LiCl·H_2_O) and lithium sulfate monohydrate (Li_2_SO_4_·H_2_O), which were analytical reagents supplied by Wuhan Xinshenshi Chemical Technology Co., Ltd., Wuhan, China. Distilled water was used and the water to cement ratio (w/c) was 0.5. The amount of Li_2_CO_3_ used as an accelerator in engineering is usually 0.4–0.6%, so the proportions of Li_2_CO_3_ in this study were 0, 0.2%, 0.4%, 0.6% and 0.8% by weight, then the contents of LiCl and Li_2_SO_4_ were calculated by maintaining the same concentration of Li^+^. So, the concentrations of Li^+^ were 0, 0.05, 0.11, 0.16 and 0.22 mmol/g of cement. A 0.3% by weight percent of borax (Na_2_B_4_O_7_·10H_2_O) (Wuhan Xinshenshi Chemical Technology Co., Ltd., Wuhan, China) was added into CSA cement mixtures, which acted as a retarder, avoiding the extremely rapid solidification of CSA cement mixtures when casting the specimens. In this work, Li_2_CO_3_, LiCl and Li_2_SO_4_ were denoted by “CO_3_”, “Cl” and “SO_4_” in figures, and the five concentrations of Li^+^ were marked from numbers 0 to 4. Cement pastes mixing was performed in a standardized laboratory mixer at a medium speed for three minutes, then poured into the molds whose sizes were 40 mm × 40 mm × 40 mm. The specimens were demolded after curing in the air for 8 h, and then stored in a standard curing room (20 ± 2 °C and relative humidity 98%) to 24 h and 28 days for testing.

### 2.2. Test Methods

(1) Compressive Strength

Cubic compressive strength test was conducted to investigate the strength variation of specimens at the age of 8 h, 24 h and 28 days. The size of cubic specimens were 40 mm × 40 mm × 40 mm. The loading rate of cubic compressive test was 0.4 MPa/s.

(2) Thermogravimetric (TG) Analysis

The hydration process of samples was ceased at the predetermined time (8 h, 24 h and 28 d) by successively immersing these samples into isopropanol for 24 h, and then these samples were ground into powder with particle sizes smaller than 100 μm and dried in vacuum oven at 50 °C. TG analysis was performed to study thermal decomposition properties of hydrated mineral phases of CSA cement pastes by SDT Q600 thermogravimetric analysis instrument (TA Instruments) (Waters Technology (Shanghai) Co., Ltd., Shanghai, China). The rate of high-purity nitrogen, which acted as protective gas, was 100 mL/min, the initial temperature was 20 °C with a heating rate of 10 °C/min and the final temperature was 1000 °C.

(3) X-ray Diffraction (XRD) Analysis

Crystallized phases were identified by XRD analysis (Panalytical X’Pert PRO MPD, Shanghai Spectris Instrument System Co., Ltd., Shanghai, China) on powder samples of CSA cement pastes at 8 h, 24 h and 28 d. A scanning speed of 2 °/min with a step size of 0.02° was used to examine the samples over the range of 5–80°.

(4) Hydration Heat Analysis

The thermodynamic and hydration characteristics of CSA cement pastes in 3 days were investigated by a multichannel calorimeter (TA instruments) (TAM Air, Waters Technology (Shanghai) Co., Ltd., Shanghai, China) of the isothermal heat conduction type operated at 20 ± 0.02 °C. The weight in this test was about 5.0 g.

(5) Scanning Electron Microscope (SEM) Observation

Field emission SEM (Zeiss SIGMA 300) was applied to investigate the surface morphology of CSA cement pastes at 8 h and 28 days. The resolution of test machine was 1.3 nm (20 kV), and the accelerating voltage was 20 kV. In this study, the sample were cut into thin sheets (about 5 mm × 5 mm × 1.5 mm) after ceasing the hydration process, and then dried in the vacuum oven at 50 °C prior to SEM observation.

## 3. Results

### 3.1. Compressive Strength

**Figure 1:**
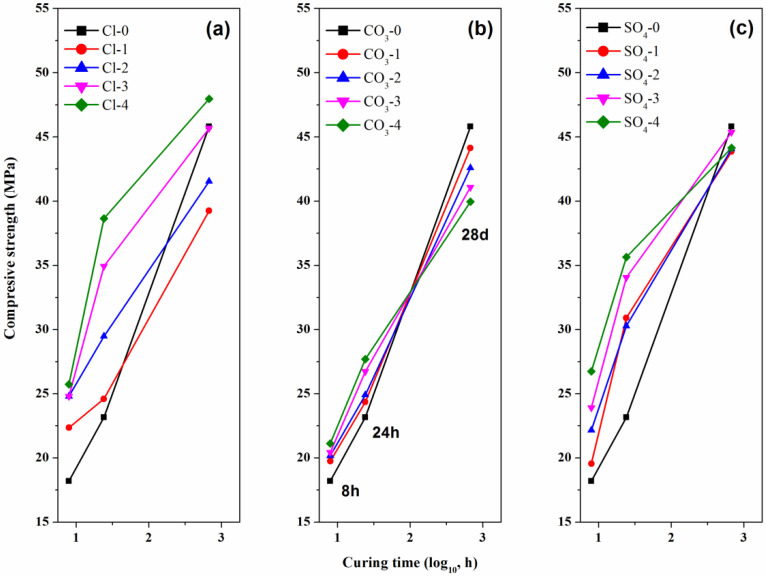
Compressive strength of CSA cement pastes as a function of curing time. (**a**) LiCl, (**b**) Li_2_CO_3_ and (**c**) Li_2_SO_4_.

The compressive strengths of CSA cement pastes are shown in Figure 1. It is seen that the early compressive strengths of 8 h and 24 h increased with the dosages of lithium compounds for all of samples. However, the larger content of Li_2_CO_3_, the lower compressive strength at 28 d, which was contrary to those of 8 h and 24 h. It is in accordance with the results in Wang’s and Juilland’s studies [19,20]. The early hydration acceleration caused by Li_2_CO_3_ hindered the subsequent hydration of CSA cement minerals to a certain extent. For the CSA cement pastes mixed with large concentration of LiCl and Li_2_SO_4_, the compressive strength of 24 h can be higher than 30 MPa. Although the early strength was relatively high, the increase rate of later strength (28 d) became lower with the addition of lithium compounds. The later compressive strength of lithium-free CSA cement paste at 28 days can overtake that of lithium-containing pastes, and the later strengths of CSA cement pastes mixed with different contents of Li_2_SO_4_ were very close, indicating that lithium compounds have no effect on the later hydration process. Comparing the compressive strengths of CSA cement pastes mixed with three different lithium compounds respectively at the same hydration time, it was revealed that all the three lithium compounds could effectively enhance the early compressive strength, especially before 8 h. Besides, the compressive strengths of the samples with the addition of LiCl or Li_2_SO_4_ were higher than that of Li_2_CO_3_, which indicated that the accelerating effect of LiCl and Li_2_SO_4_ was stronger than Li_2_CO_3_.

### 3.2. Hydration Heat Analysis

**Figure 2:**
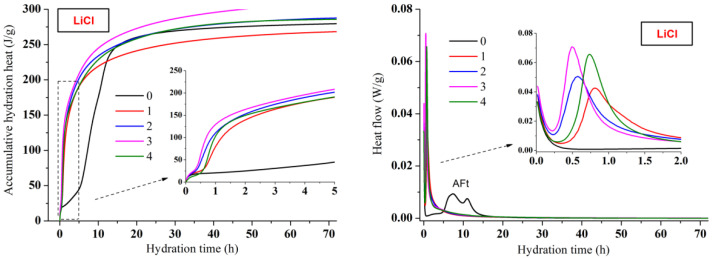
Influence of LiCl on hydration heat releasing behavior of CSA cement.

**Figure 3:**
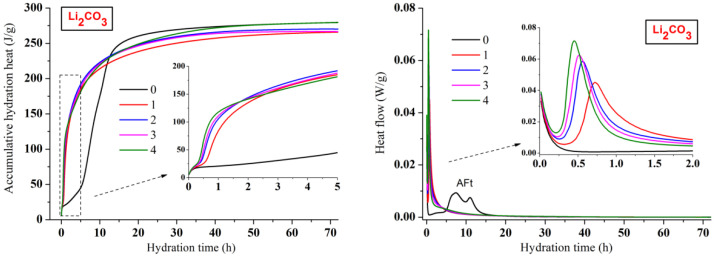
Influence of Li_2_CO_3_ on hydration heat releasing behavior of CSA cement.

**Figure 4:**
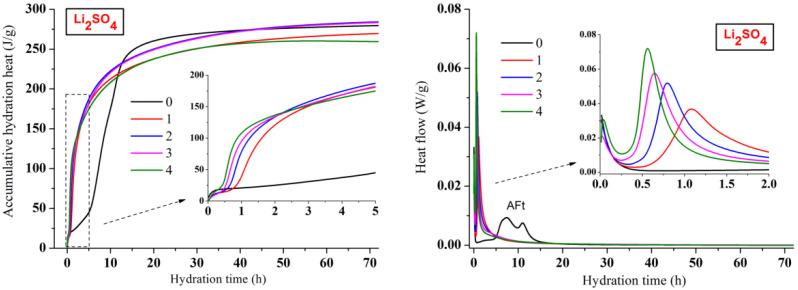
Influence of Li_2_SO_4_ on hydration heat releasing behavior of CSA cement.

The early accumulative hydration heat and heat flow curves of CSA cement pastes with three lithium compounds respectively are shown in Figure 2, Figure 3 and Figure 4. The one-day hydration process of CSA cement (black curves) is similar to that of ordinary Portland cement [30,31]. Once mixing water with cement, there would be an initial peak on the heat flow curve, which was attributed to the dissolution of CSA cement particles. However, the initial peaks were not exhibited in this study, because the dissolution happened in a very short time, which was too rapid to be recorded by the device. Then came the hydration induction period. When the induction period ended, it entered the hydration acceleration period and gradually transitioned to the hydration deceleration period. However, unlike ordinary Portland cement, CSA cement showed two consecutive exothermic peaks during the hydration acceleration period, which was led by different AFt formation stages.

As shown in Figure 2, Figure 3 and Figure 4, the hydration process of CSA cement pastes with the addition of lithium compounds is extraordinary different from the one without Li^+^. The hydration process of CSA cement strongly accelerated by three lithium compounds, and the heat flow curves presented only one sharp exothermic peak rather than two peaks compared to the pastes mixed with only water. It suggested that the hydration induction period of CSA cement disappeared due to the utilization of lithium compounds. The heat flow peaks of CSA cement pastes with lithium compounds almost emerged in one hour. However, the first peak of CSA cement paste without Li^+^ appeared after 7 h. For the CSA cement pastes mixed with Li_2_CO_3_ (Figure 3) or Li_2_SO_4_ (Figure 4), the higher contents of lithium compounds contributed to the larger heat flow rate and accumulative hydration heat at the beginning of hydration, representing the stronger accelerating effect. However, the pattern was reversed after 12 h and the accumulative hydration heat of the CSA cement paste without Li^+^ became higher. It is shown that although lithium compounds accelerated the early hydration process and enhanced the early compressive strength, the subsequent hydration process was delayed. This is consistent with the fact that the compressive strength of CSA cement paste without Li^+^ at 28 days was equal or even higher than those of the CSA cement pastes mixed with lithium compounds. For the CSA cement pastes mixed with LiCl (Figure 2), the variation of hydration heat was almost similar to Li_2_CO_3_ or Li_2_SO_4_ except Cl-4 group whose content of LiCl was the highest. This indicated that the LiCl content should be below 0.8% of CSA cement by weight. Comparing the early accumulative hydration heat of CSA cement pastes with the addition of three lithium compounds, it can be found that the accelerating effect of LiCl or Li_2_SO_4_ was more effective than that of Li_2_CO_3_, resulting in higher compressive strength.

### 3.3. TG Analysis

**Figure 5:**
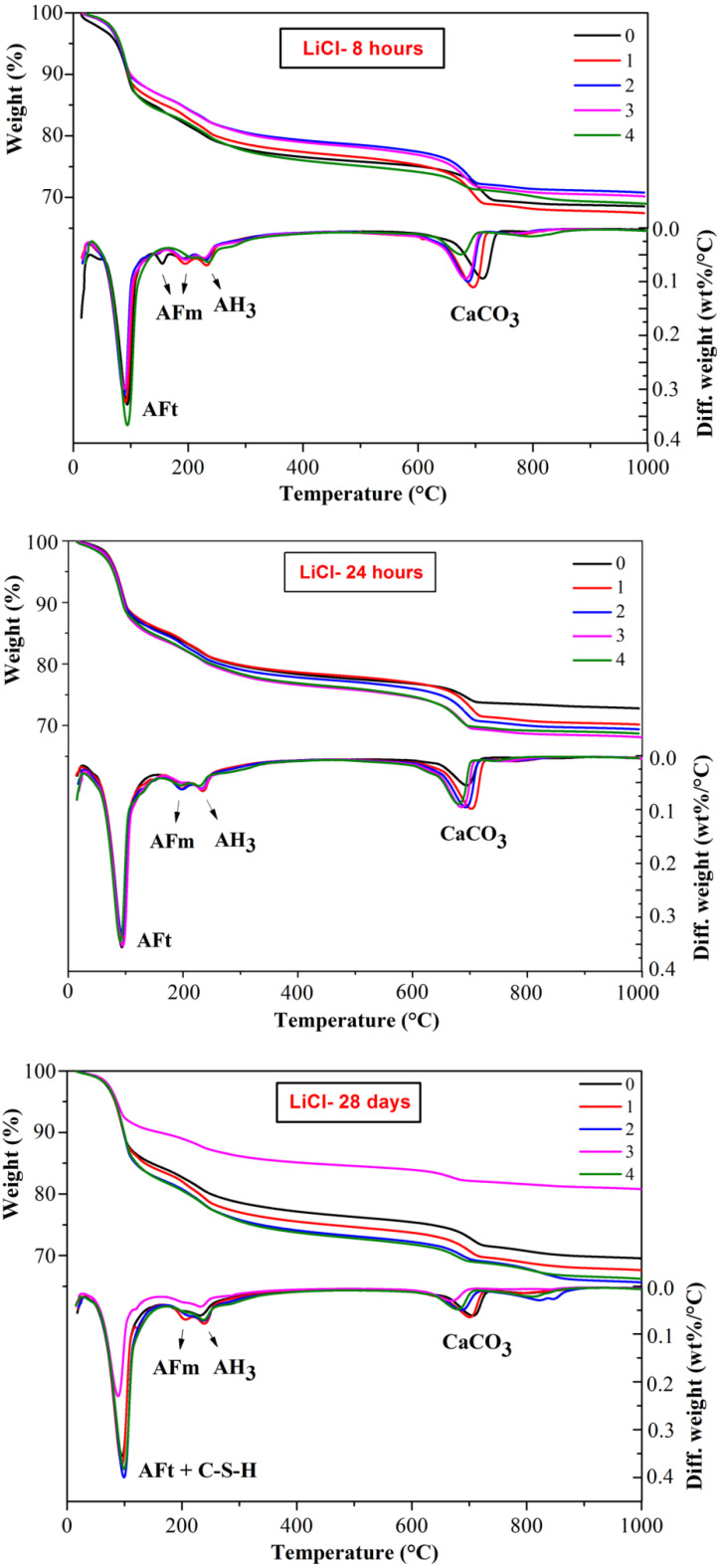
Influence of LiCl on thermal decomposition behaviors of CSA cement pastes.

**Figure 6:**
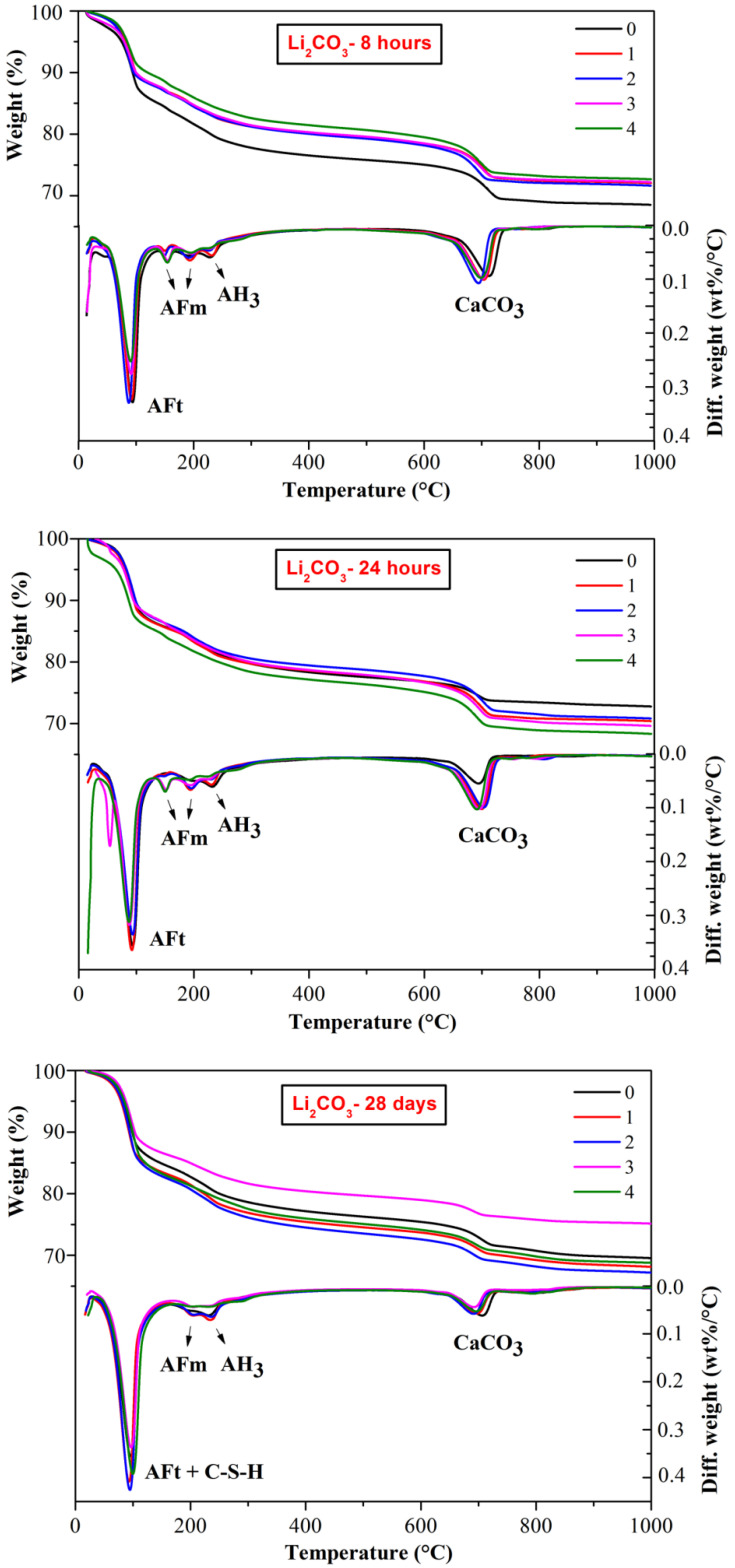
Influence of Li_2_CO_3_ on thermal decomposition behaviors of CSA cement pastes.

**Figure 7:**
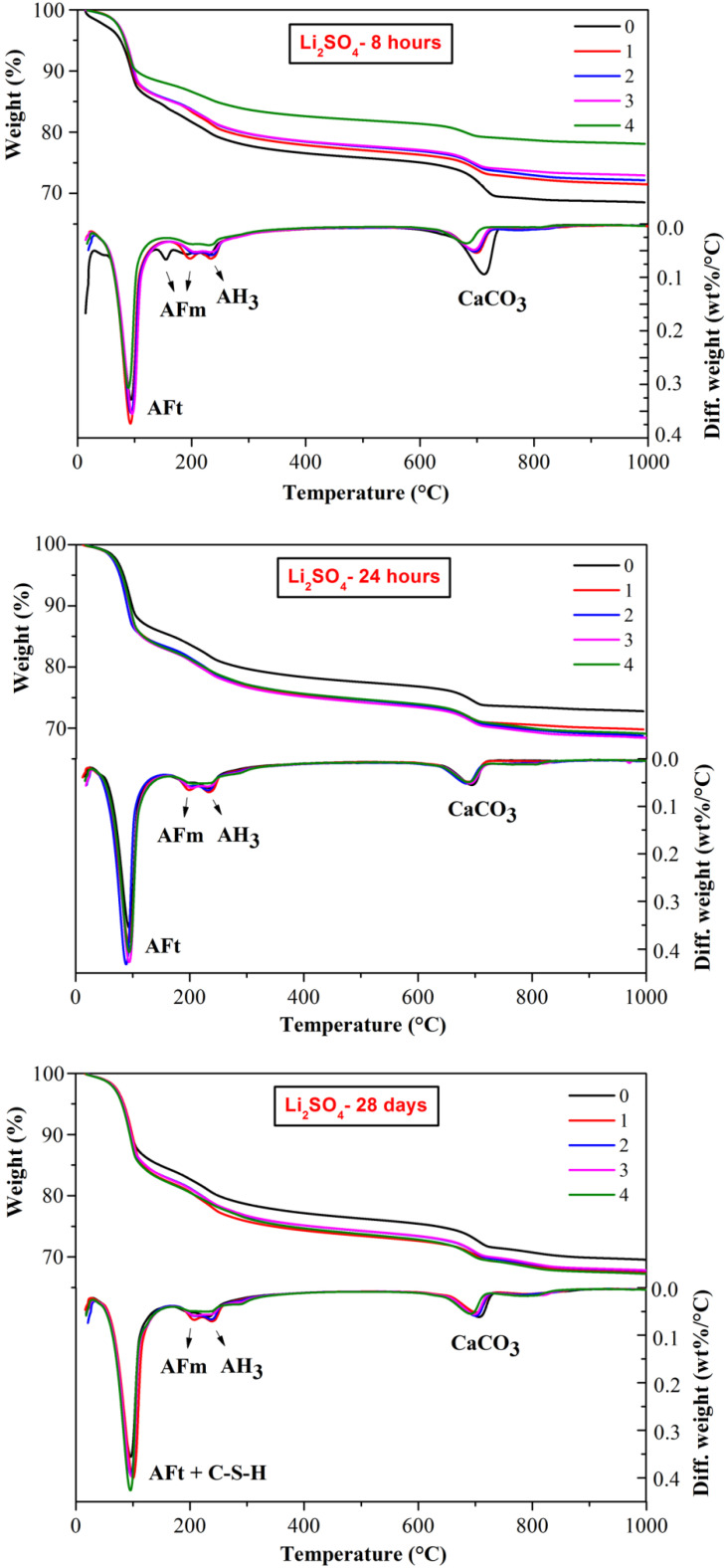
Influence of Li_2_SO_4_ on thermal decomposition behaviors of CSA cement pastes.

The thermal decomposition behavior of CSA cement pastes with the addition of three lithium compounds at a selected hydration time are shown in Figure 5, Figure 6 and Figure 7, respectively. The weight loss between 50 and 150 °C is mainly caused by dehydration of AFt [32]; the weight loss between 150 and 200 °C is due to the dehydration of AFm phases; the weight loss between 200 and 250 °C is the dehydration of AH_3_ [27] and the weight loss between 600 and 800 °C is attributed to decomposition of CaCO_3_ [33]. The dehydration of C-S-H also contributes to a certain amount of weight loss and partially overlapped with the AFt peaks. Since C-S-H is mainly produced by the slow hydration of C_2_S, the weight loss at 28 d might be relatively more significant.

As shown in Figure 5, Figure 6 and Figure 7, a large amount of AFt formed together with some AFm phases and AH_3_ during the hydration process of CSA cement. The amount of lithium compounds added had little influence on the type of hydration products. It is worth noting that some AFm phases formed at early hydration period, 8 h and 24 h, corresponding to the endothermic peak of approximate 150 °C, however, the peaks of AFm phase disappeared at 28 d, indicating that AFm may transform into AFt or other hydration phases at later hydration. Interestingly, for 24 h hydration, there were obvious endothermic peak of AFm phase (150 °C) at DTG curves of CSA cement pastes mixed with Li_2_CO_3_ (Figure 6) but no peaks found at the curves of LiCl and Li_2_SO_4_, suggesting that LiCl and Li_2_SO_4_ were more effective on the acceleration of the early hydration process.

In the decomposition temperature ranges of 50–150 °C and 600–800 °C, the weight loss is listed in Table 2 and Table 3, respectively. From the TG figures and tables, it can be found that the weight loss corresponding to AFt (including C-S-H) increased with the hydration time. In addition, the weight loss of CSA cement pastes mixed with lithium compounds was higher than the pastes without Li^+^, a large content of lithium compounds contributed to more weight loss, illustrating the forming of more AFt. According to Table 2, the weight loss of CSA cement pastes mixed with LiCl was higher than that of the pastes mixed with Li_2_CO_3_, but lower than that of the pastes mixed with Li_2_SO_4_. Therefore, different anions have different accelerating effects on the hydration process of CSA cement. As for Table 3, corresponding to the decomposition of CaCO_3_, the weight loss of CSA cement pastes mixed with Li_2_CO_3_ was the highest, which can be explained by the introduction of CO_3_^2^^−^, and the weight loss of the CSA cement pastes mixed with LiCl or Li_2_SO_4_ was closer to that of pure CSA cement pastes as the hydration time increased.

### 3.4. XRD Analysis

**Figure 8:**
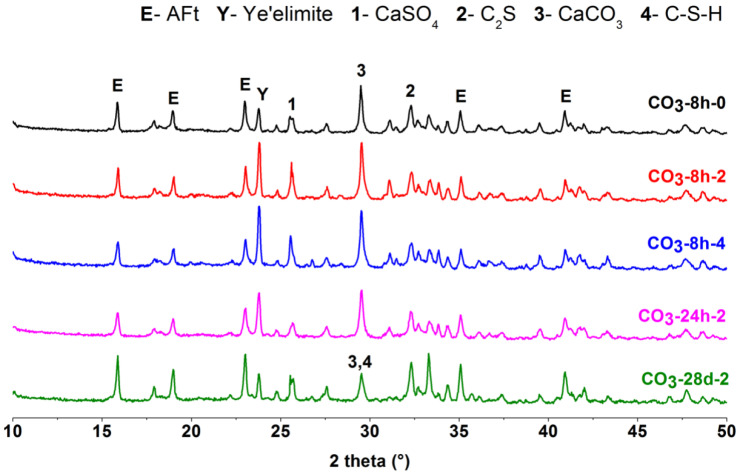
XRD patterns of CSA cement pastes mixed with Li_2_CO_3_.

**Figure 9:**
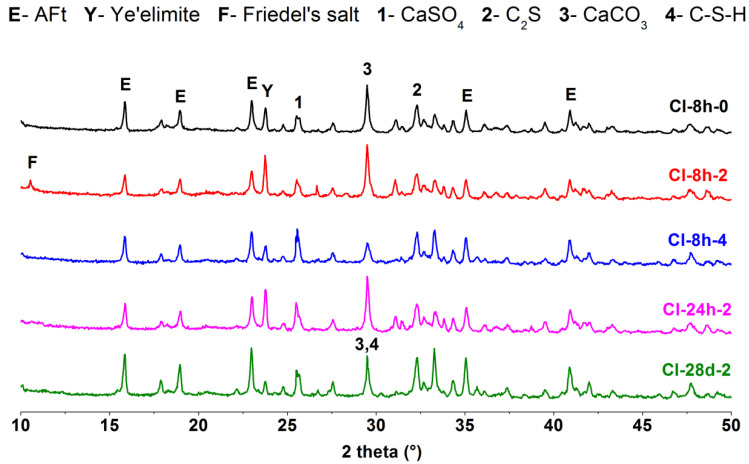
XRD patterns of CSA cement pastes mixed with LiCl.

**Figure 10:**
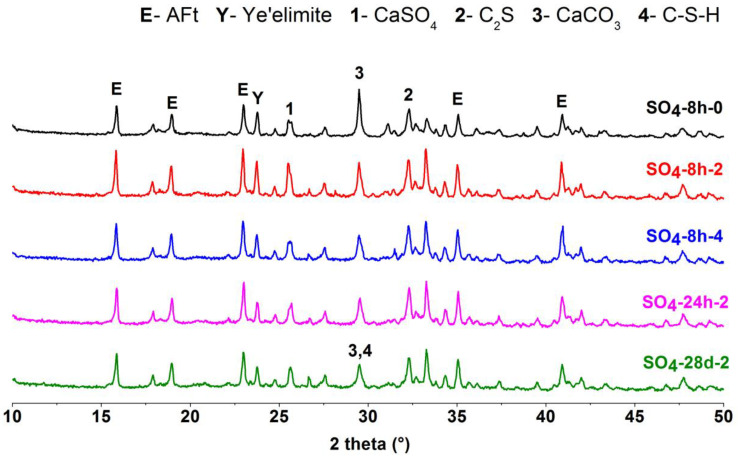
XRD patterns of CSA cement pastes mixed with Li_2_SO_4_.

The hydrated products of the CSA cement pastes mixed with different lithium compounds at different ages were characterized by XRD. The results of CSA cement pastes mixed with Li_2_CO_3_ are shown in Figure 8, and the results of the pastes mixed with LiCl and Li_2_SO_4_ are displayed in Figure 9 and Figure 10, respectively. For each lithium compound, only three Li^+^ concentrations of No. 0, 2 and 4 (0, 0.11 and 0.22 mmol/g) were selected for comparison. Among them, the role of lithium compound in the hydration process of CSA cement was analyzed with the Li^+^ concentration of No. 2 as the research object.

As shown in Figure 8, Figure 9 and Figure 10, the composition of CSA cement pastes was AFt, ye’elimite, CaSO_4_, C_2_S, CaCO_3_ and C-S-H. Lithium compounds have little effect on the type of hydration products, but it is clear that the addition of LiCl caused the occurrence of Friedel’s salt (Figure 9) in early hydration products. Since the same ordinate range was fixed, the intensity of diffraction peaks can roughly explain the content change of the same hydration product, thereby reflecting the hydration degree of CSA cement. At 8 h, Li_2_SO_4_ has an obvious acceleration effect on the early hydration of CSA cement, so the peak intensity of AFt was generally higher. It can be seen from the comparison that only Li_2_CO_3_ did not cause a decrease in the peak intensity of CaCO_3_. For the pastes with Li_2_CO_3_ and Li_2_SO_4_, the hydration products of 8 h and 24 h were almost unchanged; while for the paste with LiCl, the diffraction peak of Friedel’s salt disappeared at 24 h, and even no longer appeared at 28 d. After 28 days of hydration, the CSA cement pastes mixed with three lithium compounds continued to produce more AFt, and the peak intensity of ye’elimite decreased compared with that at 8 h and 24 h, but the difference between them was small, which suggested that three lithium compounds have little influence on the later hydration process of CSA cement. The figures also showed that the peak intensity of C_2_S was not significantly reduced, and subsequent hydration may be dominated by C_2_S, generating more C-S-H.

### 3.5. SEM Analysis

**Figure 11:**
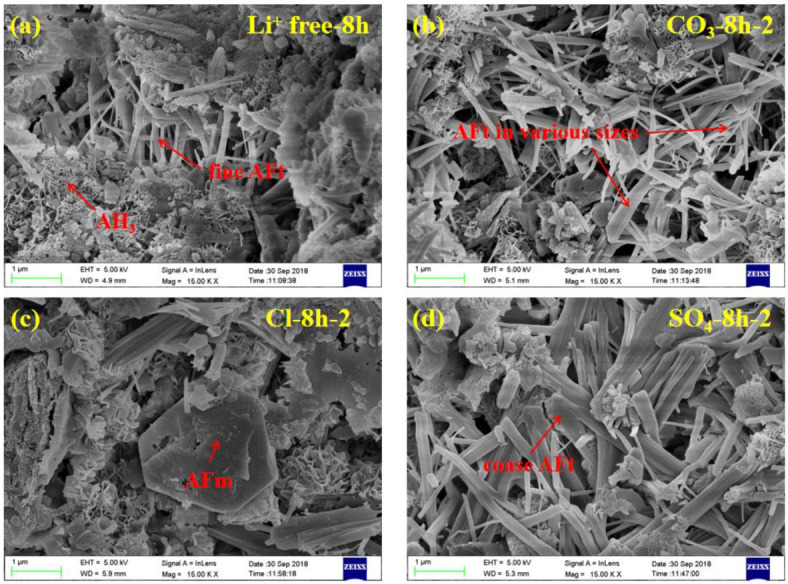
SEM images of CSA cement pastes mixed with different lithium compounds at 8 h. (**a**) Li^+^ free, (**b**) Li_2_CO_3_, (**c**) LiCl and (**d**) Li_2_SO_4_.

**Figure 12:**
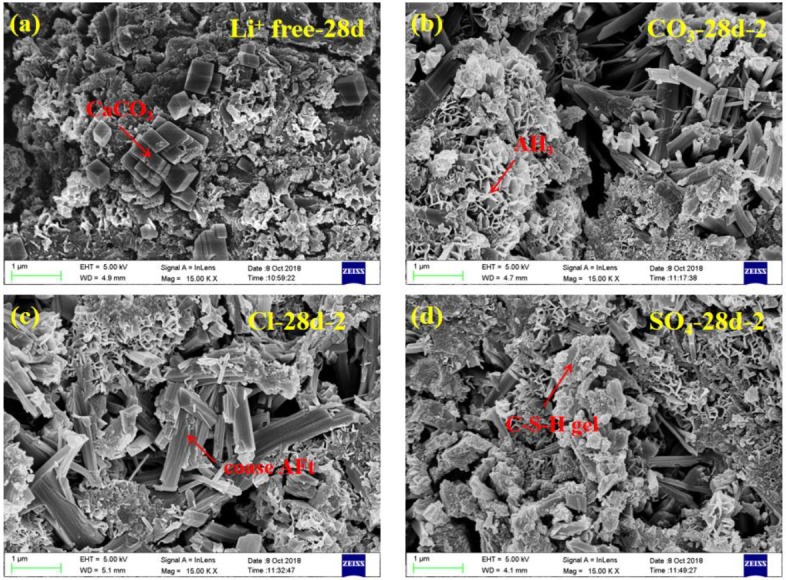
SEM images of CSA cement pastes mixed with different lithium compounds at 28 days. (**a**) Li^+^ free, (**b**) Li_2_CO_3_, (**c**) LiCl and (**d**) Li_2_SO_4_.

The SEM images of CSA cement pastes mixed with three lithium compounds at 8 h and 28 d are shown in Figure 11 and Figure 12, respectively. The magnification was fixed at 15K×. Since the contents of three lithium compounds have little effect on the type of CSA cement hydration products, the groups with the same Li^+^ concentration of No. 2 were selected for characterization.

From the Figure 11a, there were micron-scale needle-like AFt crystals, agglomerated lamellar AH_3_ and some unhydrated phases in CSA cement pastes at 8 h. Compared with the controlled group, the addition of Li_2_CO_3_ can accelerate the generation of AFt, thus some large-sized AFt can be found mixed with the small particles, as shown in Figure 11b. The acceleration effect of Li_2_SO_4_ was more obvious, and the AFt of coarse grains was almost covered in Figure 11d. Except for the large-sized AFt, only the plate-like AFm phase can be observed in Figure 11c in the LiCl group, which may be related to Friedel’s salt.

At 28 d (Figure 12), the microstructure of CSA cement pastes became denser than that of 8 h, and the differences of accelerating effect between three lithium compounds almost disappeared. There were many hydration products covering on the surface of AFt, including cubic CaCO_3_, C-S-H gel particles [34], etc. The AFm phase of the LiCl group no longer appeared. These SEM results are consistent with the test results shown above.

## 4. Discussion

### 4.1. The Influence of Li^+^ on Hydration of CSA Cement

According to the results above, Li^+^ could significantly accelerate the early hydration of CSA cement, but the difference of the accelerating effect between different content of Li^+^ was not particularly distinct. Since the accelerating effect may level off when the concentration of Li^+^ is higher than 0.03 mmol/g of cement [19,27,28], and the lithium concentrations in this work were all larger than 0.03 mmol/g of cement, the accelerating effect of Li^+^ was close. During the hydration process of CSA cement, lithium compounds would react with hydrated CH and form LiOH, significantly increasing the alkalinity of hydration environment around cement particles. Simultaneously, OH^−^ accelerates the dissolution of Al^3+^ by substituting the water molecules around Al^3+^, reducing the free energy of nucleation and the critical size of crystal nucleus, thus it is beneficial to the formation of octahedral [Al(OH)_6_]^3−^, which is the critical process of AFt formation. Besides, as a strong base, LiOH ionizes completely in water, Li^+^ can form a tetra-coordinate structure by incorporating with OH^−^ and facilitate the polymerization of octahedral [Al(OH)_6_]^3−^. Therefore, the hydration process of CSA cement can be significantly accelerated by the lithium compound.

### 4.2. The Influence of the Lithium Compound Anion on Hydration of CSA Cement

It shows that all three lithium compounds could significantly accelerate the early hydration of CSA cement. However, under the same concentration of Li^+^, the compressive strength and early hydration characteristics of CSA cement pastes were different because of different anions. Li_2_CO_3_ reacted with hydrated CH and induced the precipitation of CaCO_3_, making the microstructure denser, so that the compressive strength of CSA cement pastes slightly increased. The reaction can be represented as Equation (4).

(4) $$Li_{2}CO_{3}+CH\to 2LiOH+CaCO_{3}$$

For LiCl, there are two existential states of Cl^−^ in cement pastes, that is, the free state and solidification state. The solidification of Cl^−^ is chemically coordinated with aluminum phases, such as the AFm phase. Once mixing LiCl with CSA cement, Cl^−^ can transform the hydrated AFm phase in the CSA cement pastes into Friedel’s salt by swapping out the SO_4_^2−^ of the AFm structure. It seems to be conducive to produce more AFt, but more Ca^2+^ need to be consumed. The reaction can be represented as Equations (5) and (6). Under the same concentration of Li^+^, the compressive strength and hydration heat of CSA cement pastes mixed with LiCl is higher than that with Li_2_CO_3_, but the AFt content is not very high.

(5) $$2Cl^{-}+AFm\to SO_{4}{}^{2-}+Freidel’s\cdot salt$$

(6) $$3SO_{4}{}^{2-}+3Ca^{2+}+2AH_{3}+3CH+26H\to AFt$$

When adding Li_2_SO_4_ into CSA cement pastes, SO_4_^2−^ reacts with CH and generate active calcium sulfate. Then the active calcium sulfate can react with hydrated AH_3_ and CH to promote the formation and growth of AFt, so there are many large-sized AFt in the Li_2_SO_4_ group. To sum up, the accelerating effect of Li_2_SO_4_ is stronger than that of Li_2_CO_3_ but close to that of LiCl. The reaction process can be represented as Equations (7) and (8).

(7) $$Li_{2}SO_{4}+CH+2H\to 2LiOH+C\bar{S}H_{2}$$

(8) $$3C\bar{S}H_{2}+2AH_{3}+3CH+20H\to AFt$$

## 5. Conclusions

In this work, three kinds of lithium compounds (Li_2_CO_3_, LiCl and Li_2_SO_4_) could all significantly accelerate the hydration process of calcium sulfoaluminate (CSA) cement. The difference of the accelerating effect between different contents of Li^+^ was not very distinct. Although the early hydration acceleration of CSA cement was mainly attributing to Li^+^, the anions of three different lithium compounds also played important roles.

1. The introduction of CO_3_^2−^ could improve the precipitation of CaCO_3_ for denser microstructure.
2. The introduction of Cl^−^ might accelerate the hydration process by facilitating the transformation of AFm phase into Friedel’s salt and swapping out SO_4_^2−^ required for the AFt formation.
3. The introduction of SO_4_^2−^ might increase the content of active calcium sulfate, which further reacted with AH_3_ and CH produced by hydration to promote the growth of AFt.

According to the test results, the accelerating effect of LiCl and Li_2_SO_4_ was stronger than that of Li_2_CO_3_, but the difference of accelerating degree between LiCl and Li_2_SO_4_ was not very distinct. However, it should be considered that Cl^−^ might cause corrosion of steel bars when using LiCl as a super early strength agent in engineering. As for Li_2_SO_4_, it could better promote the early strength development of CSA cement without obvious adverse durability problems.

## Figures and Tables

**Table 1:** Chemical composition of CSA cement (wt %).

SiO_2_	Al_2_O_3_	Fe_2_O_3_	CaO	MgO	K_2_O	Na_2_O	SO_3_	TiO_2_	CO_2_	Total
6.03	18.77	2.44	47.56	1.75	0.77	0.08	16.22	0.9	5.11	99.63

**Table 2:** Thermal weight loss between 50 and 150 °C.

**Curing Time**	**Lithium Compounds**	**Thermal Weight Loss/%**				
		0	1	2	3	4
8 h	LiCl	11.7	12.7	11.6	11.7	14.6
	Li_2_CO_3_	11.7	11.0	10.1	9.3	9.1
	Li_2_SO_4_	11.7	13.4	13.1	13.5	13.8
24 h	LiCl	13.1	13.1	13.4	14.4	13.4
	Li_2_CO_3_	13.1	12.7	12.5	12.9	13.0
	Li_2_SO_4_	13.1	15.7	15.0	15.5	15.6
28 d	LiCl	13.9	14.7	15.9	15.5	15.6
	Li_2_CO_3_	13.9	15.2	16.1	14.9	15.8
	Li_2_SO_4_	13.9	16.3	15.5	15.6	15.7

**Table 3:** Thermal weight loss between 600 and 800 °C.

**Curing Time**	**Lithium Compounds**	**Thermal Weight Loss/%**				
		0	1	2	3	4
8 h	LiCl	4.8	4.2	4.6	4.5	5.0
	Li_2_CO_3_	4.8	6.6	6.7	6.6	6.6
	Li_2_SO_4_	4.8	3.9	4.0	3.5	3.7
24 h	LiCl	5.1	4.8	4.7	5.3	5.5
	Li_2_CO_3_	5.1	6.3	6.3	6.4	6.2
	Li_2_SO_4_	5.1	3.3	4.0	4.0	4.0
28 d	LiCl	4.7	4.9	4.3	3.4	4.1
	Li_2_CO_3_	4.7	5.9	6.1	6.0	6.5
	Li_2_SO_4_	4.7	4.1	4.6	4.5	4.6
